# ACID: annotation of cassette and integron data

**DOI:** 10.1186/1471-2105-10-118

**Published:** 2009-04-21

**Authors:** Michael J Joss, Jeremy E Koenig, Maurizio Labbate, Martin F Polz, Michael R Gillings, Harold W Stokes, W Ford Doolittle, Yan Boucher

**Affiliations:** 1 Department of Biological Sciences, Macquarie University, Sydney, NSW 2109, Australia; 2 Department of Biochemistry and Molecular Biology, Dalhousie University, Halifax, NS, B3H 1X5, Canada; 3 Department of Medical and Molecular Biosciences, University of Technology, Sydney, NSW 2007, Australia; 4 Department of Civil and Environmental Engineering, Massachusetts Institute of Technology, Cambridge, MA 02139, USA

## Abstract

**Background:**

Although integrons and their associated gene cassettes are present in ~10% of bacteria and can represent up to 3% of the genome in which they are found, very few have been properly identified and annotated in public databases. These genetic elements have been overlooked in comparison to other vectors that facilitate lateral gene transfer between microorganisms.

**Description:**

By automating the identification of integron integrase genes and of the non-coding cassette-associated *attC *recombination sites, we were able to assemble a database containing all publicly available sequence information regarding these genetic elements. Specialists manually curated the database and this information was used to improve the automated detection and annotation of integrons and their encoded gene cassettes. ACID (annotation of cassette and integron data) can be searched using a range of queries and the data can be downloaded in a number of formats. Users can readily annotate their own data and integrate it into ACID using the tools provided.

**Conclusion:**

ACID is a community resource providing easy access to annotations of integrons and making tools available to detect them in novel sequence data. ACID also hosts a forum to prompt integron-related discussion, which can hopefully lead to a more universal definition of this genetic element.

## Background

**Figure 1:**
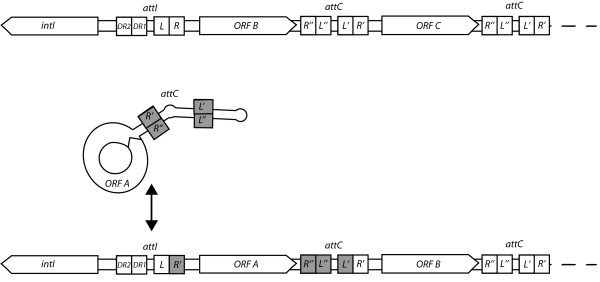
**General structure of the integron/gene cassette system**. This representation is consistent with the single-strand cleavage model proposed by MacDonald et al., 2006 [3]. The secondary structure illustrated for part of the free gene cassette (center) is achieved through base-pairing of the bottom DNA strand within the *attC*-encoded imperfect repeats (L', L" and R', R"). The IntI binding sites in *attI *known to be required (L, R) and accessory (DR1, DR2) for *attC *× *attI *recombination are also illustrated [16,17]. The diagram is based on functional studies of class 1, class 3 and *Vibrio *integrons and might not apply to all integron types.

Integrons were discovered about two decades ago as a result of their role in the evolution of multi-drug-resistant bacteria [1,2]. These genetic elements can perform acquisition, rearrangement and expression of genetic material that is part of gene cassettes. Gene cassettes are one of the simplest known mobile elements. They comprise gene(s) associated with a recombination site most commonly referred to as *attC *[3] and less commonly as 59-base elements (59-be) [4]. The integron captures gene cassettes through site-specific recombination carried-out by the encoded tyrosine recombinase (IntI). These captured cassettes are most commonly inserted by this recombination activity at the integron attachment site (*attI*) [4] (Figure 1). Such capture events can occur repeatedly and, in the case of some chromosomal integrons, this process can lead to the creation of large arrays encoding hundreds of gene cassettes [5]. A promoter, P_c_, often located upstream from *attI*, is thought to enhance expression of proximal cassette-associated genes in some integrons [6]. The ability to capture disparate individual genes and physically link them in arrays suitable for co-expression is a trait unique to this genetic element. The result is an assembly of functionally interacting genes theoretically facilitating the rapid evolution of new phenotypes [7].

Initially, integrons were thought of as specialized elements mostly involved in the accumulation of gene cassettes encoding antibiotic resistance determinants in pathogenic bacteria [8]. The advent of genomics and the availability of numerous genome sequences from environmental bacteria made it clear that the integron is a more ancient and widespread gene capture system [9]. Despite the fact that this genetic element is found in about 10% of all sequenced genomes and that cassette arrays can be as large as 150 kb [10], few integrons have been properly identified and annotated as such. Even when the integron integrase gene is annotated due to its sequence similarity to characterized homologs, the gene cassettes associated with it are labeled as simple open reading frames (ORFs). This is because *attC *sites, the most distinctive feature of gene cassettes, are non-coding regions and therefore, not recognized by standard automated genome annotation pipelines.

Integrons are a flexible and fast-evolving part of microbial genomes, and their associated cassette arrays represent a unique and segregated gene pool (the genes they carry are rarely found outside these genetic elements). However, because integrons (other than the specialized variants carrying antibiotic resistance genes) rarely have a detectable phenotype under laboratory conditions, they are seldom emphasized in genomic studies. Their proper identification and annotation could help us understand their role in microbial adaptation.

We have created ACID, which stands for Annotation of Cassette and Integron Data, as a resource for the biology community. ACID contains all integrons and gene cassette sequences available in public databases manually curated and accurately annotated. Users can freely access and download these data and/or automatically annotate and submit their own sequences. Tools for the visualization and comparison of cassette arrays are also made available.

## Construction and Content

MySQL 5.0 is used as the database engine and a PHP 5.2 front end using the YUI set of AJAX tools is used for display and manipulation of the data [11-13]. The database forum uses phpBB [14]. Design of the database relies on two major tables: 'Sequence' and 'Interest'. 'Sequence' contains data pertaining directly to the acquisition of the original DNA sequence. 'Interest', on the other hand, contains details related to the annotation of that sequence. These tables form a one-to-many relationship.

### Definition of integron/gene cassette system components

The mission of ACID is to provide a consistent annotation framework for the description of the integron/gene cassette system by providing researchers with a platform to exchange their ideas as they pertain to this genetic element. For the initial construction of the database; we have developed basic definitions for the different elements of integrons: the integron-encoded integrase (*intI*), recombination sites *attI *and *attC*, gene cassettes, gene cassette open reading frames (CassORFs) and more generally, ORFs. The database however, has been built so it can easily be updated if scientific evidence suggests that modification to the definition of various integron components is required.

The *intI *is the sequence region that encodes or may have encoded (in the case of degenerate *intIs*) the integron integrase gene. Integron integrases, part of the tyrosine recombinase family, are different from other members of that protein family insofar as, they encode an additional functional domain required for integron gene cassette site-specific recombination [15-17]. In this initiative, integron integrases are identified by their sequence similarity to the previously characterized homologs in ACID. This comparison is made using BLASTX [18].

The *attI *region has only been defined functionally in a limited number of integron classes [19]. It is most commonly located upstream from the *intI *gene and ends with the 7 bp *attI *core site (GTTRRRY) at the start of the first inserted cassette (R in Figure 1) [19-21]. Due to the lack of experimental data regarding the precise boundary of the *attI *site at the end closest to *intI *(in most cases), we define this feature as the region starting immediately upstream from the start/stop codon of the integrase gene (depending on its orientation with respect to the integrated gene cassettes) and ending with the first detectable 7 bp core site (R).

For the purpose of this database, the *attC *site comprises inner repeats L' and L", the variable region between these repeats, and the flanking R' and R" repeats (Figure 1). *attCs *are defined here in accordance with experimental data that has revealed the site of cassette integration [22]. The unit of transfer, the gene cassette, comprises one *attC *and in most instances a single ORF. Specifically, we define a single gene cassette as the DNA-sequence region from the first 'T' of the R' repeat-region within a given *attC*, up to and including the first 'G' of the next, downstream R'. Therefore, the *attC *site that "belongs" to a given cassette is the sequence of the element as it appears in the circular form (Figure 1). We are using the L'/L" and R'/R" nomenclature for naming the *attC *imperfect repeats corresponding to the IntI-binding domains instead of the 1L/2L and 1R/2R system. This is to be consistent with the terminology used in the recent description of the gene cassette insertion/excision mechanism by the integron integrase [3,23].

CassORFs are defined as any ORF located in the intervening region between an *attI *site and an adjacent *attC *site or, two adjacent *attC *sites. Some allowance for overlap with these enclosing *attI *and *attC *sites is made with at most 15 bp of overlap of the ORF and an *attC/attI *site being the upper limit.

### Integron/gene cassette system annotation procedure

ACID's automated annotation tool first identifies the *intI *gene and then analyzes the neighboring sequence for the presence of *attC *like motifs. Two strategies (described in the algorithm section) are used to detect *attC *sites and the site must match the user-adjustable cut-off restrictions for both methods (described below) to be annotated as such. Given our definition of gene cassettes (above), the latter are automatically annotated based on the identified *attCs*. By default, the largest ORF located within each cassette, if it encodes a predicted product greater than seventy amino acids, is annotated as a CassORF.

We have also provided the user with the option of searching for ORFs in any given DNA sequence regardless of its nature. Therefore, such a search would retrieve all ORFs associated with a given integron sequence -IntIs, CassORFs and ORFs flanking the integron.

The results obtained at each of these stages can be inspected and edited by the user if desired. This is especially useful if the sequence proves to be more complex than usual (e.g. multiple *intI *genes are present in the sequence, extremely degenerate *attC *regions are known to exist or large insertion regions are located within the cassette array).

### Data sources for ACID

The database has been compiled from a number of sources. The core of these data is manually annotated sequences identified as containing integrons according to the scientific literature [10]. These sequences were used to compile a list of known *intI *genes. The amino acid sequences of these IntI proteins were then used to perform a similarity search against the NCBI's non-redundant nucleotide sequence database with TBLASTN [18]. Any records that had a high degree of similarity to the previously identified *intI *genes were then added and automatically annotated. Unpublished sequence data from ongoing studies (genomic and metagenomic) was manually entered to complement the Genbank records.

### Compilation of statistics on ACID content

Various statistics were compiled on the database content. The diversity of gene cassettes found in the database was evaluated using the EstimateS 8.0 statistics software package [24]. For the analyses, we arbitrarily defined cassette-types as any two or more cassettes that have at least a 150 nucleotide overlap with a specified DNA identity. Cassette nucleotide identities of 70, 80, 90, 95, 97 and 99 percent were considered in the analyses.

Gene cassettes were functionally annotated with the Meta Genome Rapid Annotation using Subsystem Technology (MG-RAST) server [25]. This website provides a SEED-based environment that allows users to upload metagenomes for automated analyses [26]. In MG-RAST, novel sequences are screened for potential protein encoding genes via a BLASTX search against the SEED comprehensive non-redundant database (sourced from all major public databases). This similarity search allows the association of a novel sequence with a SEED subsystem, which represents a specific metabolic function.

### Algorithms for the identification of *attC *sites

This section provides an overview of the general methodology used in the two approaches implemented within the database used for the identification of *attC *sites. These two methods are, String search and Feedback loop.

#### String search identification of *attC *sites

**Figure 2:**
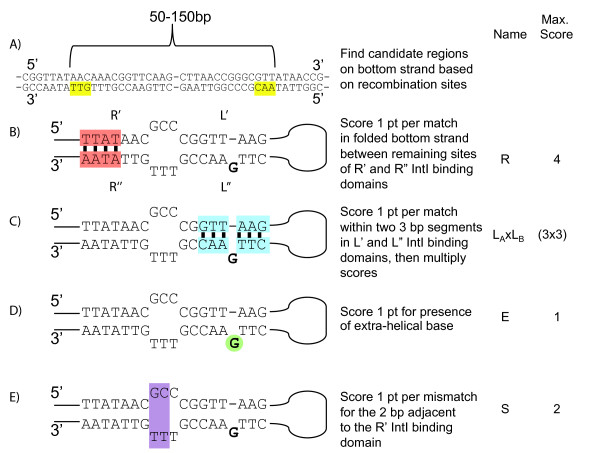
**Logistics of String search for the identification of *attC *sites**. **(A) **Search for the nearly invariant nucleotides of the inverse core (AAC) and core (GTT) sites on the bottom DNA strand (TTG and CAA) **(B) **Assign 1 point per match between the flanking repeats of the *attC *sites (R' and R"), excluding the three nearly invariant nucleotides. **(C) **Assign 1 point per match between the inner repeats of the *attC *sites (L' and L"). **(D) **Score 1 point for the presence of the typical extra-helical base, defined here as E and, **(E) **Score 1 pt for each mismatch in the 2 bp adjacent to the R' repeat referred to as site S. Final scores are divided by the total possible score and are reported as percentage values.

Cassette-associated *attC *recombination sites have several defining features, the most conserved of these being the core and inverse core sites (R' and R", Figure 1), and modifying the sequence of these sites can dramatically affect recombination activity [27]. For our first approach, we considered a string-based search algorithm in order to identify conserved nucleotides in the *attC *sequence. Initially, a set of genome sequences for which the integrons and gene cassettes had been manually annotated was used as target sequences for testing. These sequences were used to search for the nearly invariant nucleotides of the inverse core (AAC) and core (GTT) sites, separated by a distance typical of previously identified *attC *sites (50 to 150 bp) (Figure 2A).

This method, however, statistically produces a large number of false positives, a 3 bp motif like AAC appears once every 64 bases (P(N) = 0.25 and P(N)^3 ^= 0.15625 or, 1/64). To filter out false positives from the group of sequences that encoded the inverse core/core sites, we scored them by assessing other features commonly found in *attC *sites [22]. The total score is composed of several individual categories that reflect separate structural components typical of *attC *sites. These structural components play a role in creating the single (bottom) strand folded conformation required for integrase-mediated recombination (Figure 2). Specifically, we assign 1 point per match between flanking repeats (core/inverse core sites) of the *attC *site (R' and R"). The total possible score of this property referred to as R, is 4 (Figure 2B). In addition, we score 1 point per match between the inner repeats of the *attC *site (L' and L"). We made the decision to weight these base-pairing events to make them more meaningful. Specifically, we considered the potential secondary structure of this simple site as two 3 bp regions. These properties, referred to as L_A _and L_B_, when multiplied, produce a maximum score of 9 (Figure 2C). These are multiplied so that pairing is required in each of these two regions to produce a score. For example, L_A _= 3 and L_B _= 0 would produce a score of 0 (3 × 0). However, if L_A _and L_B _each had 2 base-pairing events, the score would be 4.

In addition, we score 1 point for the presence of the typical extra-helical base, defined here as E and, and 1 pt for each mismatch in the 2 bp adjacent to R' referred to as site S (Figure 2D and 2E, respectively). Final scores are divided by the maximum possible total score and are reported as percentage values. We defined this cut off at 75%, which results in the overall lowest rate of false positives and false negatives. However, the user has the option to define his/her own cut off which may be more appropriate for a specific dataset.

#### Feedback loop identification of *attC *sites

The second strategy for *attC *identification uses a position weight matrix methodology. The idea here is to train the scoring algorithm so that it "learns" which components of the sequence are important for *attC *identification. All *attC *sites that had been annotated manually, in addition to a curated collection of *attC *sites identified using the search string strategy (defined above) were compiled and used to build the position weight matrix. The nucleotide frequencies at the 25 terminal positions at either end of the 2766 positive *attC *sites were considered since these encode the repeats required to form the IntI binding sites (R"/R' and L"/L' in Figure 1). The probability of a particular nucleotide occurring at each of the 25 terminal positions was calculated from these data. If the nucleotide in question was not important to the function of the *attC *site (i.e. did not facilitate secondary structure formation) we assumed that the probability of any given nucleotide observed to occur at this position would be close to random (25%). Each nucleotide position was compared to its binding partner within the *attC *site. If they base paired, a score of 1 was recorded. A score of 0 was recorded for a mismatch. Base pairing 3 positions up and down stream from each location was also considered. From these data, the probability of a particular base to pair with a base at the same position at the opposite end of the *attC *could be calculated. The percentage weightings were then linearly adjusted to consider the GC content of the sequence. Nucleotides that occurred more often than random frequencies were positively weighted and, conversely, nucleotides that present close to random frequencies were negatively weighted. Percentage weightings were then summed up for each nucleotide position across all known *attC *sites. The result is an algorithm that has "learned" which nucleotides should be considered important to integron *attC *sites when searching sequence space.

As new *attC *sites are identified, they add to the collection of positive examples, thus affecting the weightings for future *attC *sites. Therefore, as *attC *functional information grows, so does the accuracy of the feedback loop scoring method. It is important to stress however, that the user has the option to accept or reject the proposed *attC *sites identified by this algorithm. Rejecting false positives will ultimately lead to a more accurate dataset that is used to train the algorithm. Therefore, we propose that this method be used in combination with meticulous attention to detail on the part of the user. Also, it is possible that this algorithm may become too specific. For example, there is presently only a limited diversity of *attCs *in the database and therefore, an algorithm trained on this potentially biased dataset may not detect new, diverse *attC*s. For this reason, the user may choose a minimum score of zero for this method of cassette identification, making it possible to identify novel *attC *sites by the string search method alone.

## Utility and Discussion

This section presents a brief description of the ACID database content and its usage. Detailed tutorials and instructions for the various functions of the database are available on the website.

### Entry of new data in ACID

Novel data can be added to the database by manually entering the sequence information and accompanying details into a form. This form can be invoked from the 'add' option of the 'edit' scroll-down menu found at the top of any database entry form. Alternatively, a sequence and all its associated annotations can be imported directly from Genbank (the 'import' option in the 'edit' menu). This option is especially useful if the integron sequence is part of a complete or partial genome sequence.

### Sequence annotation procedure

The database allows automatic annotation of any integron found in a proposed sequence. This is achieved in a three steps process. These may be performed individually (so the user can have input on the results, accepting or rejecting them manually) or all at once (using the 'Auto' option from the 'Annotate' drop-down menu). Annotation can only be performed on sequences entered by the user, not ones already present in the database.

#### IntI annotation

The user must choose 'IntI' from the 'Annotate' drop-down menu. The proposed data entry is then used as a query sequence for the BLASTX algorithm [18] which compares the query to all IntI protein sequences identified previously. The region of the proposed sequence that produces a significant bit score is annotated as IntI.

#### *attC *annotation

The user must choose '*attC*' from the 'Annotate' drop-down menu. The user has the option of choosing how to annotate the *attC *sites. Specifically, *attC *sites are identified using one or both strategies described in the algorithm section above. Cassettes are consequently annotated based on the positions of the *attC *sites (i.e. they are defined as the nucleotide sequence between *attC *sites).

#### ORF annotation

The user must choose 'ORF' from the 'Annotate' drop-down menu. ORFs present in the proposed sequence can be annotated based on the following criteria (none or all of which can be selected by the user): present within gene cassettes (CassORFs); present within a specific portion of the sequence (nucleotide coordinates); displaying with standard or alternative start codons; those larger than a specified minimal amino acid length.

### Database search

The user can either browse the database or perform more sophisticated searches. Using the browse option (from the drop-down 'Browse' menu) provides the user with the option of searching the database according to pre-defined categories (accession number, host species, sequence length, origin, etc). The database also encompasses extensive hierarchical search features. Two search modes (from the scroll-down 'Search' menu) are available to locate integron-related data, 'Sequence' and 'Interest'. The 'Sequence' search allows the user to retrieve complete sequence entries matching the search criteria selected. The 'Interest' search can be used to retrieve any annotated integron component (*intI*, *attC*, cassette, etc). The annotated regions/entries returned by either search mode can be downloaded in both amino acid and nucleotide FASTA format for transfer to alternative or complementary programs and further analyses.

### Discussion forum

A complete forum engine (phpBB) is included in the database to facilitate communication between scientists who are new to studying integrons or who are experienced veterans. The nature of the forum includes both general comments as they pertain to integrons, integron annotation and the gene cassette biology as well as comments specific to a particular annotation in the database. These specific annotation comments are linked back to the database so that relevant comments can be viewed for each sequence as it is being examined. We have included this option because we know that biological variation is often unpredictable and we do not propose to have accounted for all forms of integron-associated diversity. Through this open forum we hope to promote a collaborative environment promoting the sharing of integron-related knowledge.

### Overview of database content

**Figure 3:**
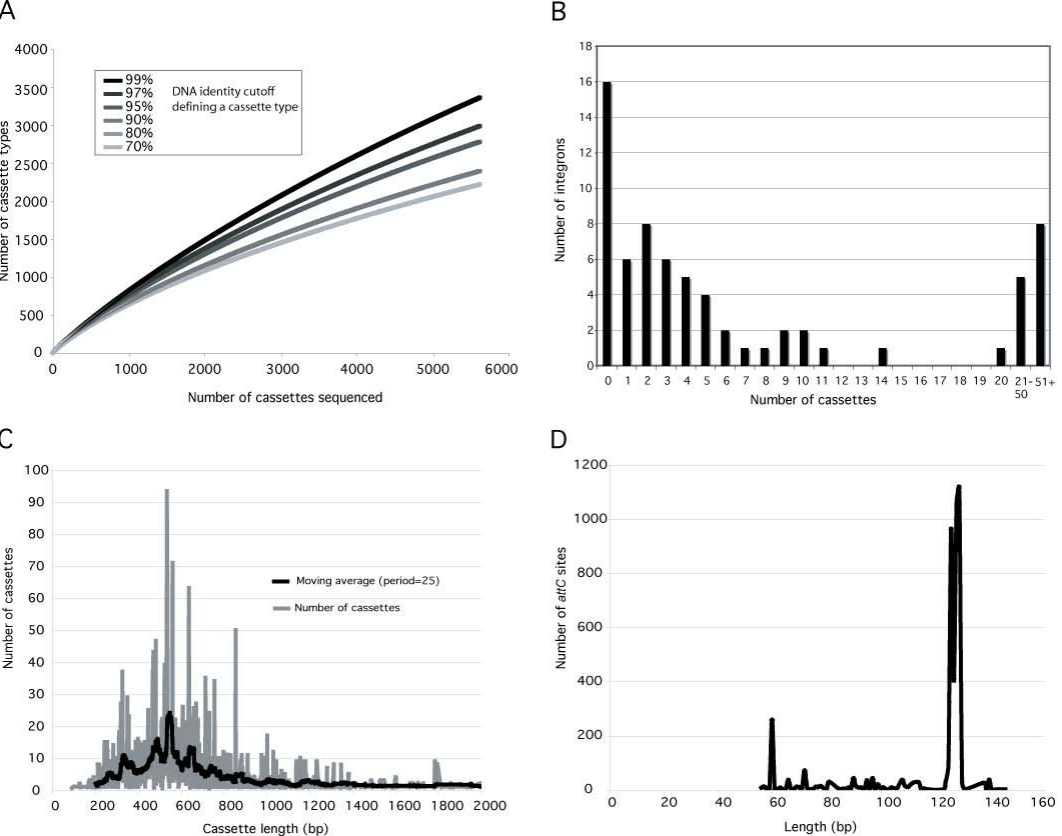
**Statistics on the various components of the integron/gene cassette system annotated with ACID**. **A) **Averaged rarefaction curves describing the diversity of gene cassette-types. **B) **Distribution of the number of cassettes encoded in different integrons. **C) **Length of gene cassettes, including their *attC *sites. **D) **Length of *attC *sites.

471 *intI *sequences and 5622 gene cassettes were collected and stored in the first version of ACID and this will be updated regularly as new data become available. Table 1 displays estimates of diversity for gene cassettes annotated in ACID. The number of cassette-types shows little variation with lowering of the DNA sequence identity cut-off below 80%, indicating a minimum of ~2200 unique cassette-types in the database (Figure 3A). This is despite important sampling bias (most of the cassettes originate from class 1 and *Vibrio *integrons), suggesting large genetic diversity in the gene cassette pool. It is obvious that the sequences currently present in ACID represent a very small portion of total cassette diversity. Indeed, a study of cassette diversity in 50 m^2 ^of forest soil estimated (very conservatively) at least 2343 cassette-types in that area [28].

**Table 1:** Diversity statistics for cassettes annotated with ACID

	DNA sequence identity thresholds defining a cassette-type (%)					
	99	97	95	90	80	70

Number of cassette-types	3354	2980	2774	2389	2213	2202
Shannon-Weiner Index	7.7	7.5	7.4	7.1	6.9	6.9
Chao-1 Estimator (Standard deviation)	8536 (198)	6827 (104)	6258 (231)	5166 (200)	4638 (182)	4638 (182)

Most integrons contain few cassettes (0–10), although some contain many (>50). These are almost always found in the marine bacterial genus *Vibrio*, with a few exceptions (such as the marine heterotroph *Saccharophagus degradans*) (Figure 3B). Most cassettes range between 200–800 bp in length, but their size can reach > 2000 bp (Figure 3C), probably due to the insertion of tranposon sequences or lysogenic phages. The *attC *sites associated with cassettes vary between sizes of 55 to 150 bp, with a significant number at 60 bp and between 126–129 bp (Figure 3D). The overrepresentation of these two sizes might be due to a sampling bias rather than functional properties, given that 60 bp is the typical length of an *attC *type common in class 1 integrons and that most *attC *sites found in *Vibrio *integrons are 126–129 bp long.

The majority (86%) of cassette ORFs did not have a homolog with an identified function, leaving a mere 14% for which a putative function could be ascribed. This is a recurring theme in both metagenomic and genomic/fosmid sequencing initiatives targeted at gene cassettes. This trend suggests an integron-specific gene cassette pool, distinct from the larger genomic gene pool. The continued accumulation of cassette sequence data will reveal the frequency at which these currently unique cassette-encoded ORFs will re-occur.

Where cassette function could be ascribed, we observed a disparate range of cassette-encoded protein functions illustrating that integrons can recruit a diversity of potentially advantageous genes (Table 2). Most notable among them are phenazine biosynthesis-like proteins and acetyletransferases. These are known virulence determinants and proteins often linked to drug resistance, respectively. Given the bias of genome sequencing initiatives toward medically relevant microorganisms, it is not surprising that the majority of ascribed cassette functions consist of those related to pathogenicity. Additional sampling of more diverse bacteria under different selection pressures should reveal a range of functions that are not appreciated by the limited sampling of integrons performed so far.

**Table 2:** Functional identification of cassette-encoded ORFs in ACID

Gene cassette annotation	Number of cassettes
Hypothetical	3059
Phenazine biosynthesis-like protein	363
Acetyltransferase	233
Plasmid maintenance system	168
3'-O-adenylyltransferase	52
Glyoxalase family protein	32
Transposase	24
Beta-lactamase (EC 3.5.2.6)	23
Isochorismatase (EC 3.3.2.1)	22
Lipoprotein Blc	22
Var1 homolog	20
Lac operon repressor type protein	19
Lactoylglutathione lyase	18
Chloramphenicol resistance protein	15
relB protein	14
Transposase OrfAB, subunit B	12
ATP-dependent Clp protease	11
Cell wall-associated hydrolases	10
DNA-damage-inducible protein J	10
Haemagglutinin associated protein	10
Microcin immunity protein MccF	10

Four bacterial phyla harbour integrons associated with gene cassettes (Proteobacteria, Chlorobi, Planctomycetes and Spirochaetes) [10]. However, the vast majority of integron diversity is found in Proteobacteria. Among the 34 different bacterial genera containing integrons and gene cassettes, 17 are from the Proteobacteria phylum. This is likely to represent a sampling bias, given that 548 out of 1047 partial or complete bacterial genome sequences publicly available represent proteobacteria.

## Conclusion

Very few integrons have been properly identified and annotated in public databases. These genetic elements have therefore been overlooked in comparison to other vectors that facilitate LGT between microorganisms. By automating the identification of integron *intI *genes and the non-coding *attC *sites associated with gene cassettes in DNA sequence data, a database has been assembled containing all publicly available sequence information regarding these genetic elements. The manually curated and publicly available web-based database can perform automated detection and annotation of integrons and gene cassettes enabling future sequence data to be incorporated easily. ACID provides an environment using consistent terms and definitions enabling simple comparison of all known examples of the integron/gene cassette system. The database can be searched using a range of queries and selected data can be downloaded in FASTA format convenient for export and integration with downstream applications. Users can readily annotate and save their own data and have the option of forwarding it to curators for addition to the main database. This database will help create more accurate annotations of bacterial genomes and facilitate a more representative sample of integron frequency and diversity.

## Availability and Requirements

ACID can be accessed at . For instructions and tutorials, click on the help menu on the toolbar at the top of the ACID website.

## Authors' contributions

MJJ created the database and the automated annotation algorithm and wrote part of the manuscript. JEK performed ecological and functional analyses of cassettes and helped with drafting and revising the manuscript. ML helped with manual curation of the annotation. YB conceived the database and drafted the manuscript. MRG helped supervise the creation of the database. WFD and HWS took part in drafting the manuscript and helped coordinate the study. MFP provided sequence data for the database and contributed to the coordination of the study.
